# Osthole protects sepsis-induced acute kidney injury via down-regulating NF-κB signal pathway

**DOI:** 10.18632/oncotarget.13592

**Published:** 2016-11-25

**Authors:** Chen Yu, Peng Li, Dong Qi, Lei Wang, Hong-lin Qu, Yue-juan Zhang, Xue-kai Wang, Hua-Ying Fan

**Affiliations:** ^1^ School of Pharmacy, Binzhou Medical University, 264003 Yantai, Shandong, P.R. China; ^2^ Department of Nephrology, Yu-Huang-Ding Hospital/Qingdao University, 264000 Yantai, Shandong P.R. China; ^3^ Yantai Food and Drug Inspection Center, 264000 Yantai, Shandong, P.R. China; ^4^ Yantai Yan-Tai-Shan Hospital, Yantai, Shandong, P.R. China; ^5^ School of Pharmacy, Key Laboratory of Molecular Pharmacology and Drug Evaluation (Yantai University), Ministry of Education, Collaborative Innovation Center of Advanced Drug Delivery System and Biotech Drugs in Universities of Shandong, Yantai University, Yantai, P.R. China

**Keywords:** osthole, sepsis, CLP, NF-κB signal pathway, acute kidney injury

## Abstract

**BACKGROUND AND PURPOSE:**

As a natural coumarin derivative from the *Cnidium monnieri(L)Cusson* fruit, osthole consists of 7-methoxy-8-isopentenoxy-coumarin. The purpose of this research is to study the mechanism and effect of osthole on sepsis-induced acute kidney injury.

**EXPERIMENTAL APPROACH:**

The protective effect of osthole on mouse macrophage RAW 264.7 and HK-2 cells induced by LPS *in vitro* and on acute kidney injury model induced by sepsis and established by puncture and cecal ligation (CLP) *in vivo* were tested.

**KEY RESULTS:**

Osthole (20, 40 mg·kg^−1^) group can greatly attenuate the changes of the score and kidney histopathology damage and enhance the survival time of septic mice. After the CLP surgery, degrees of SCr and BUN related to kidney injury were upregulated. The concentrations of SCr and BUN can be greatly reduced by treatment with osthole. Furthermore, osthole could increase bacterial killing activity and phagocytic activities of macrophages impaired after CLP partly and attenuate blood bacterial counts and leukocyte infiltration markedly. Furthermore, osthole can suppress NF-κB signal pathway through the inhibition of the nuclear translocation by regulating phosphorylation of IκBα and IKKβ and hinder the production of chemoattractant (MCP-1 and IL-8) and proinflammatory cytokines (TNF-α, IL-1β and IL-6).

**CONCLUSION AND IMPLICATIONS:**

Mainly because of its immunomodulatory properties and anti-inflammatory activity, which might be closely associated with suppression of the stimulation of the NF-κB signal pathway, osthole has protective effect on sepsis-induced kidney injury. It can be seen from such evidence that osthole can be potentially applied in the treatment of acute kidney injury.

## INTRODUCTION

During critical illness, acute kidney injury (AKI) has become more and more common in incidence, which can be related to considerable mortality and morbidity. According to the results of a number of researches, AKI rates range from 22% to 67% [1, 2] in intensive care units (ICUs) and the AKI rates of hospitalized patients range from 3.2% to 20%. In current days, supportive renal replacement therapy is the only available treatment for AKI. However, in spite of great progress in renal replacement therapies (RRTs), the prognosis of acute kidney injury patients is still poor. Statistics from clinical trials show hospital mortality rates of 58% in AKI patients who required RRT and 43% in those who did not [3].

There are a great number of causes of AKI in ill patients. As the main cause of AKI, sepsis accounts for around 50% of cases [4]. A number of researches have shown that sepsis-induced AKI is related to long-range and short-range risk of death [5]. When compared to 39% of non-septic AKI patients, mortality rate in hospitalized patients with AKI-complicating sepsis is an abysmal 70% [6].

As a natural coumarin derivative extracted from *Cnidium monnieri(L)Cusson* fruit [7], Osthole consists of 7-methoxy-8-isopentenoxy-coumarin. Study has shown that osthole shows a great number of biological and pharmacological activities, including anti-oxidation, anti-inflammatory, anti-osteoporosis, anti-tumor and anti-allergic effects and has extensive and comprehensive application in conventional Chinese medication for treatment for acute ischemic stroke [8, 9, 10, 11] and the treatment of cutaneous pruritus, eczema, sexual dysfunction, trichomonas vaginalis infection. According to the present research, osthole treatment protects murine kidney from renal I/R injury through suppressing cell apoptosis and oxidative stress [12] and protects acute lung injury induced by lipopolysaccharide [13]. Nonetheless, the mechanism and effect of osthole on AKI are not elucidated well.

Thus, the current research aims to study potential mechanisms and the effects of osthole on sepsis-induced AKI. This research shed new lights on the mechanism for the treatment of septic-AKI or AKI.

## RESULTS

### Effect of osthole in sepsis on survival time in mice

**Figure 1:**
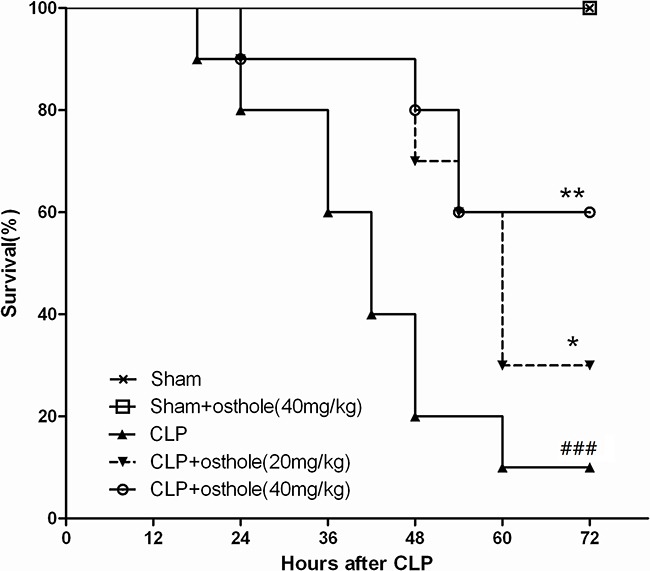
Effect of osthole on survival rate after cecal ligation and puncture (CLP) mice Mice were challenged by CLP to induce acute kidney injury with or without osthole (20 mg·kg^−1^ or 40 mg·kg^−1^) intragastrically administered after operation. Survival was monitored every 6 h during 72 h and the percent survival rate was expressed as Kaplan–Meier survival curves. *n=*10. *^###^p<* 0.001 compared to Sham group; *p<0.05 and **p<0.01 compared to CLP group.

The mortalities were higher than the sham group (*p<*0.001) after CLP during the first 72 h. On the other side, osthole decreased the death of CLP mice considerably at 40 mg·kg^−1^ (p <0.01) and 20 mg·kg^−1^ (p <0.05) of dose groups (Figure 1). Osthole did not show abnormal signs and obvious toxicity in sham+osthole (40mg·kg^−1^) group at the first 72 h. Thus, 20 mg·kg^−1^ and 40 mg·kg^−1^ osthole could be utilied in the below animal assays.

### Effects of osthole on renal injury induced by CLP

**Figure 2:**
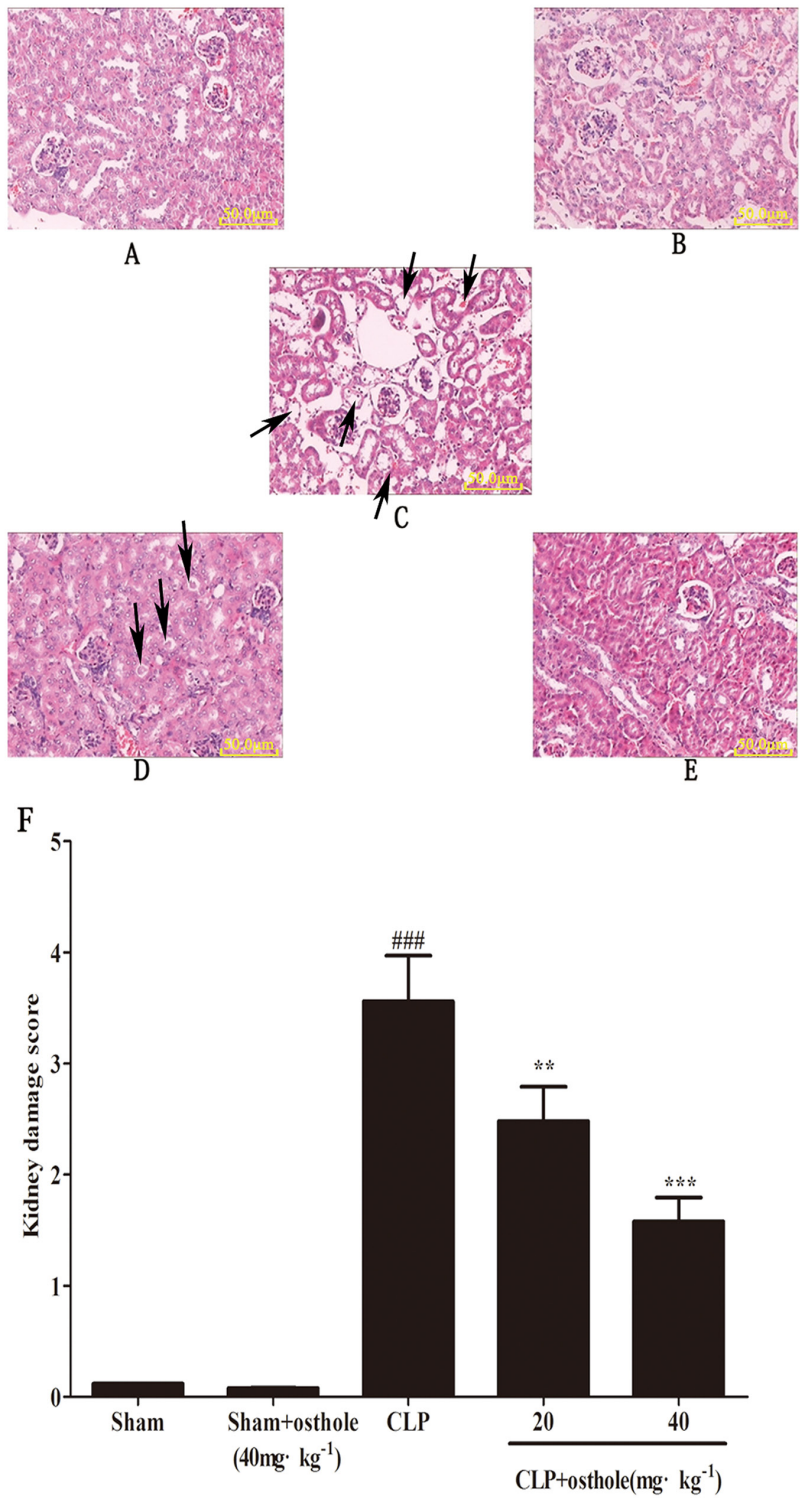
Effect of osthole on kidney injury after CLP surgery Representative histological changes in kidneys obtained from mice of different groups **A**. Sham group; **B**. Sham+ osthole group; **C**. CLP group ; **D**. CLP + osthole (20 mg·kg^−1^) group; **E**. CLP + osthole (40 mg·kg^−1^). The sections shown were harvested 24 h after CLP operation and stained with H&E. Magnification:×100. **F**. Pathological score of representative kidney samples of each group. The arrowheads indicated the representative morphological changes including tubular degeneration, necrosis and hyperemia. Data are represented as mean ± SD of 10 animals of each group. *^###^p<* 0.001 compared to Sham group, **p<0.01 and ***p<0.001 compared to CLP group.

According to Figure 2, from the morphological point of view, there was no considerable difference between the sham + osthole group and the sham group. Both were normal in appearance. Nonetheless, kidney sections from the CLP group were featured by wide degeneration in epithelial cells of renal tubules. Even severe hyperemia of renal tubule interstitial cells, cell debris concentration and severe necrosis could be seen in renal tubule lumens. These histopathologic changes could be decreased in the CLP + osthole groups in different degrees. The severity of kidney destruction was ameliorated greatly. The renal tubular epithelial cell degeneration was mild. Also, there was slight intracellular congestion and edema in the renal interstitium and renal tubule. There was a greatly decreased score of kidney pathological damage in CLP + osthole groups in comparison with that in the CLP group.

### Effects of osthole on CLP-induced renal dysfunction

**Figure 3:**
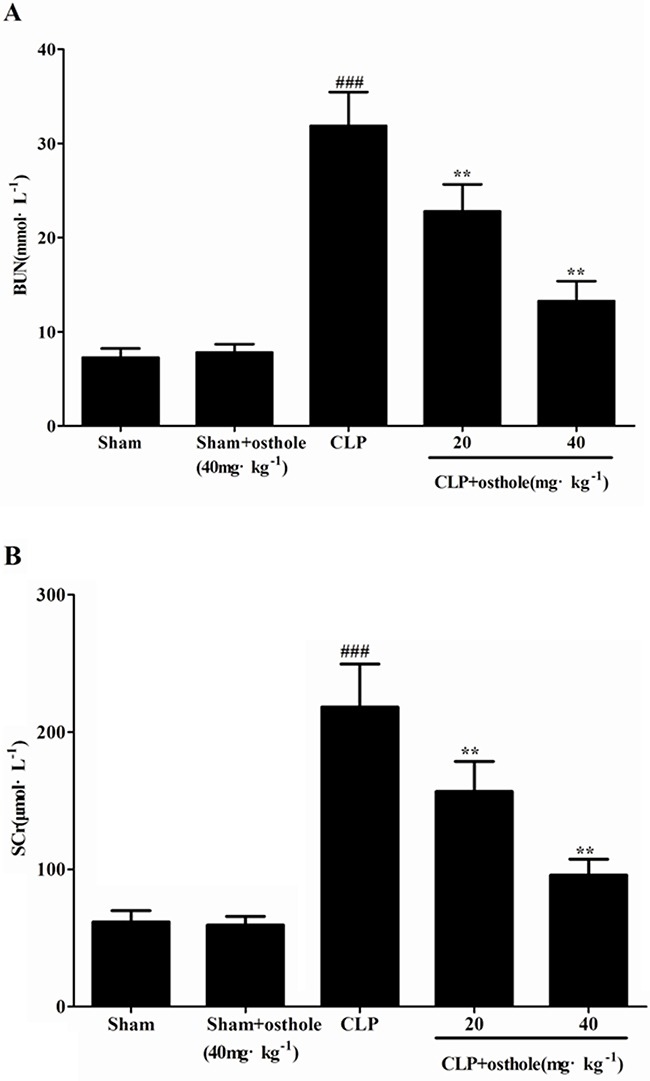
Effects of osthole on serum BUN **A**. and SCr **B**. Data are represented as mean ± SD of 10 animals of each group. *^###^p<* 0.001 compared to Sham group; **p<0.01 compared to CLP group.

As significant index of renal injury severity, SCr and BUN were utilized to assess the renal function. The increased degrees of SCr and BUN could be shown in mice suffered from CLP in comparison with sham group (*p<*0.001). Treatment with osthole (20, 40 mg·kg^−1^) reduced the degree of SCr and BUN induced by CLP. On the other hand, in sham + osthole group, only osthole administration has no considerable effect on SCr and BUN levels in comparison with that in the sham group (Figure 3A and 3B). It could be confirmed that we had built a successful animal model of sepsis-induced AKI based on the changes to serum biochemical index and renal histology. Also, the osthole has a protective effect on AKI induced by CLP without nephrotoxicity.

### Effects of osthole on inflammatory responses

**Figure 4:**
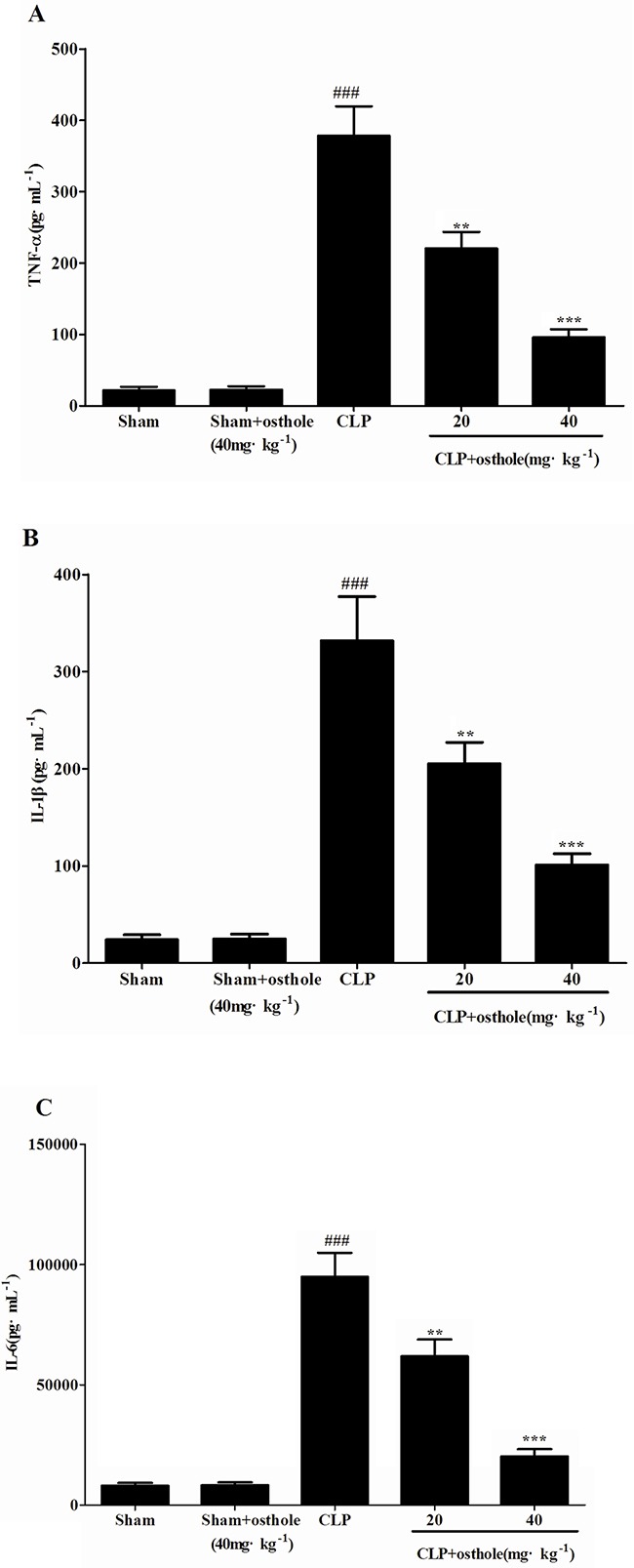
Effects of osthole on the production of inflammatory cytokines in serum from mice Quantitation of TNF-α **A**. IL-1β **B**. and IL-6 **C**. in serum was performed by ELISA. Data are represented as mean ± SD of 10 animals of each group. *^###^p<*0.001 compared to Sham group; **p<0.01 and ***p<0.001 compared to CLP group.

**Figure 5:**
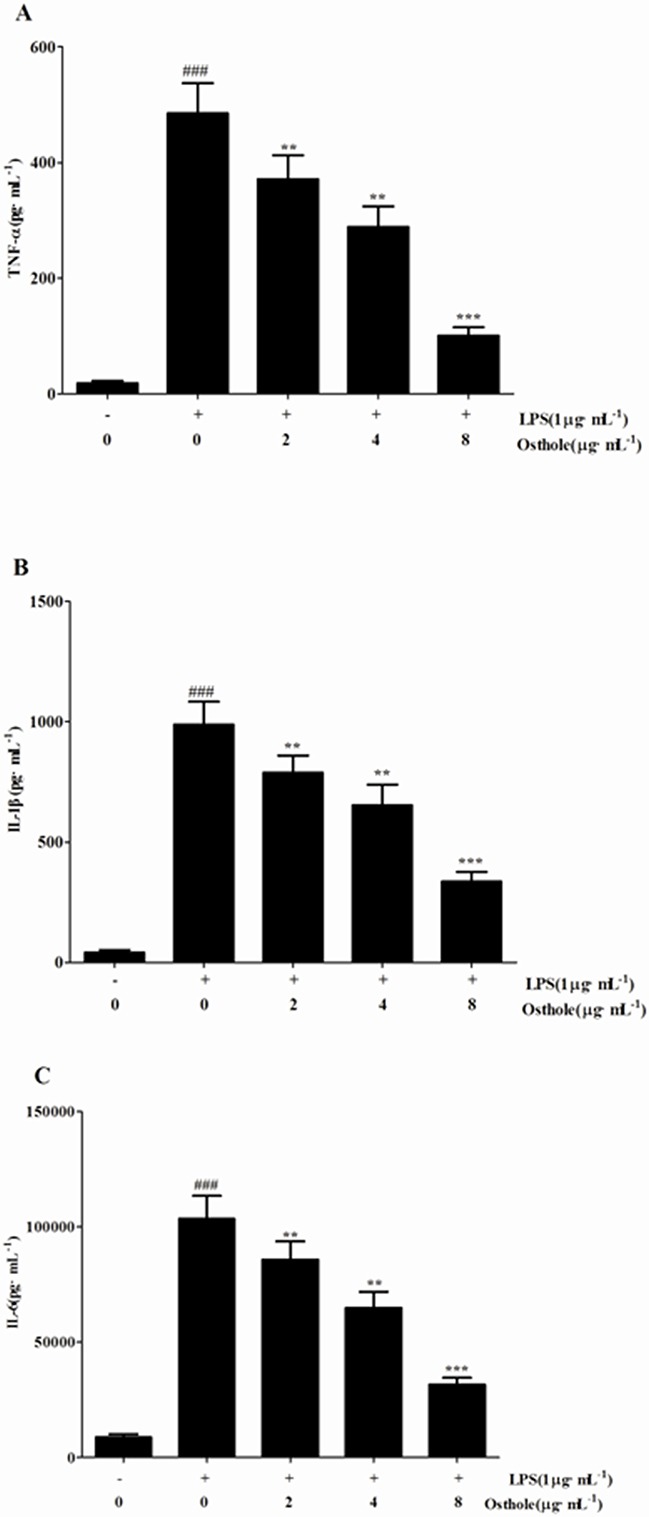
Effects of osthole on the production of inflammatory cytokines by LPS-induced RAW 264 7 cells. Quantitation of TNF-α **A**. IL-1β **B**. and IL-6 **C**. in cultural supernatants was performed by ELISA. Data are represented as mean ± SD of three independent experiments. ###*p<*0.001 compared to untreated group; **p< 0.01 and ***p<0.001 compared to LPS alone.

**Figure 6:**
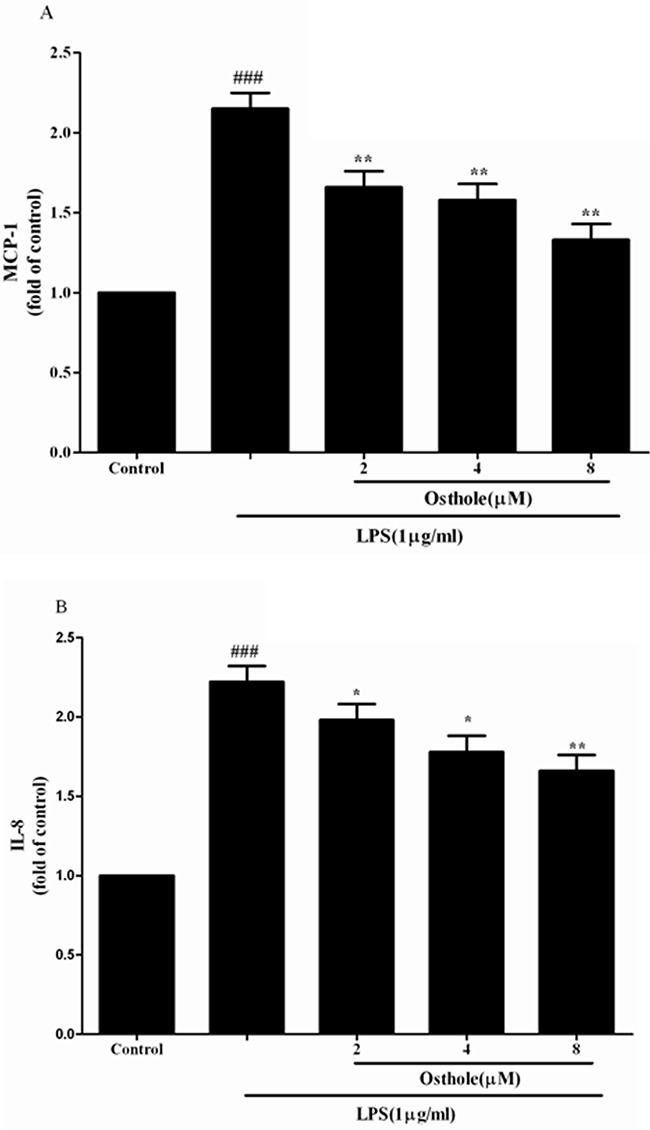
Effect of osthole on MCP-1 A. and IL-8 B. release induced by LPS in HK-2 cells Cells were treated with LPS(1 μg/mL) with or without osthole for 24 h. 100μL of culture medium in each group was taken out to measure the levels of MCP-1 and IL-8 using ELISA kits. Data are representedas mean±SD of three independent experiments. ^###^*p <* 0.001 compared to untreated group, *p < 0.05 and **p < 0.01 compared to LPS alone.

An uncontrollably sustained and vigorous inflammatory response is a characteristic of serious sepsis. Pro-inflammatory cytokines (IL-6, TNF-α andIL-1β) in our research were identified to study how osthole influenced the levels of pro-inflammatory cytokines in CLP mice and cultured the RAW cells (Figure 4 and 5). According to the results, the degrees of IL-6, TNF-α and IL-1β were comparable and low in untreated LPS RAW cells and in sham mice with osthole or vehicle. Contrast, a considerable increase degree of these pro-inflammatory cytokines could be observed in LPS-induced cells and CLP group 24 h after the surgery. The elevated degrees of IL-6, TNF-α and IL-1β were inhibited significantly by treatment with osthole in vitro and vivo. Furthermore, to inhibit the infiltration of macrophages/monocytes (Figure 6), osthole could attenuate the release of IL-8 and MCP-1in LPS-induced HK-2 cells.

**Figure 7:**
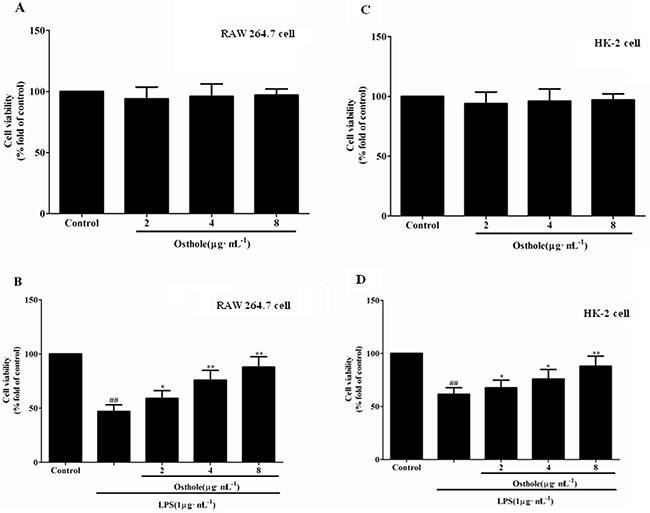
Effect of osthole on cells viability tested by MTT assay **A**. Effect of osthole on RAW 264.7 cells proliferation in normal condition. **B**. Effect of osthole on LPS-induced RAW 264.7 cells proliferation. **C**. Effect of osthole on HK-2 cells proliferation in normal condition. **D**. Effect of osthole on LPS-induced HK-2 cells proliferation. Results are expressed as percentage of viable cells when compared with control groups. Data are representedas mean±SD of three independent experiments. ^##^*p<* 0.01 compared to untreated group; *p< 0.05 and **p< 0.01 compared to LPS alone.

In addition, osthole attenuated the cytotoxicity induced by LPS in a dose-dependent manner (Figure 7B and 7D) whereas osthole at the concentrations tested in cells did not show considerable cytotoxicity as e by the MTT assay (Figure 7A and 7C). According to the statistics from *in vitro* and *in vivo*, osthole could restrict the inflammatory cytokine production and the inhibitory effects could not be described to the non-specific cytotoxic effects.

**Figure 8:**
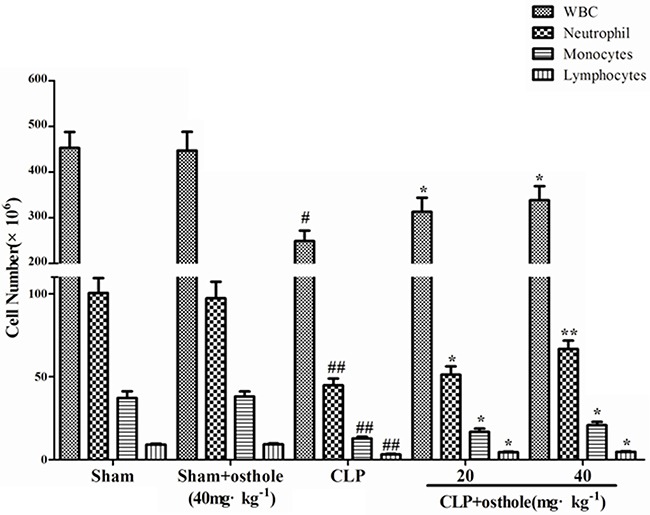
Effect of osthole on peripheral white blood cell counts in CLP-induced sepsis Blood samples were withdrawn at 24 h after the CLP surgery. Total and differential cell counts were measured. Data are represented as mean ± SD of 10 animals of each group. ^#^*p<* 0.05 and ^##^*p<* 0.01 compared to Sham group; *p< 0.05 and ** *p<* 0.01 compared to CLP group.

Peripheral WBC counts were detected to show the severity of sepsis and the migration of inflammatory cell. According to the results, 24 h after the CLP surgery, peripheral WBC count was reduced considerably. Osthole enhanced leukocyte number considerably, including neutrophil and monocytes, whereas only in sham mice, osthole treatment did not change the WBC level (Figure 8). The results demonstrated that osthole could effectively attenuate the severity of inflammatory responses induced by CLP surgery.

**Figure 9:**
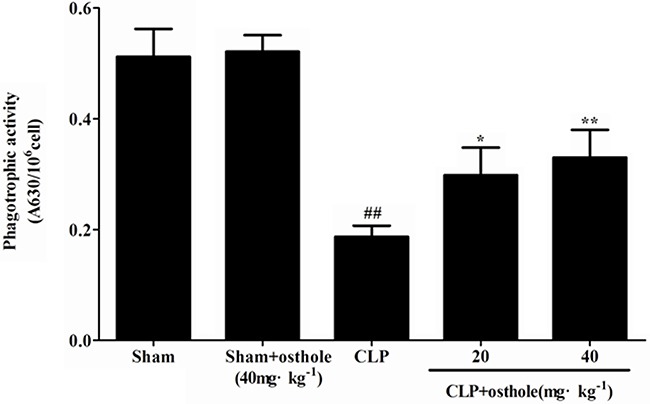
Effect of osthole on peritoneal macrophage phagocytic activity in CLP-induced mice sepsis Macrophages harvested 24 h after CLP were incubated with zymosan and NBT. Phagocytosis was measured as OD 630 nm. Data are expressed as mean ± SD. (n=10). ^##^*p<* 0.01 compared to Sham group; *p< 0.05 and ** *p<* 0.01 compared to CLP group. NBT: nitroblue tetrazolium.

To study how osthole influence the phagocytic activity of peritoneal macrophages in CLP-induced sepsis, the phagocytosis was tested. According to the results from the phagocytosis experiments (Figure 9), the phagocytic function of macrophage was suppressed in CLP mice. Osthole treatment had increased phagocytic function of peritoneal macrophages effectively. According to the results as above, because of its immunomodulatory and anti-inflammatory properties, osthole has protective effect on septic AKI.

### Effect of osthole on blood bacterial clearance

**Figure 10:**
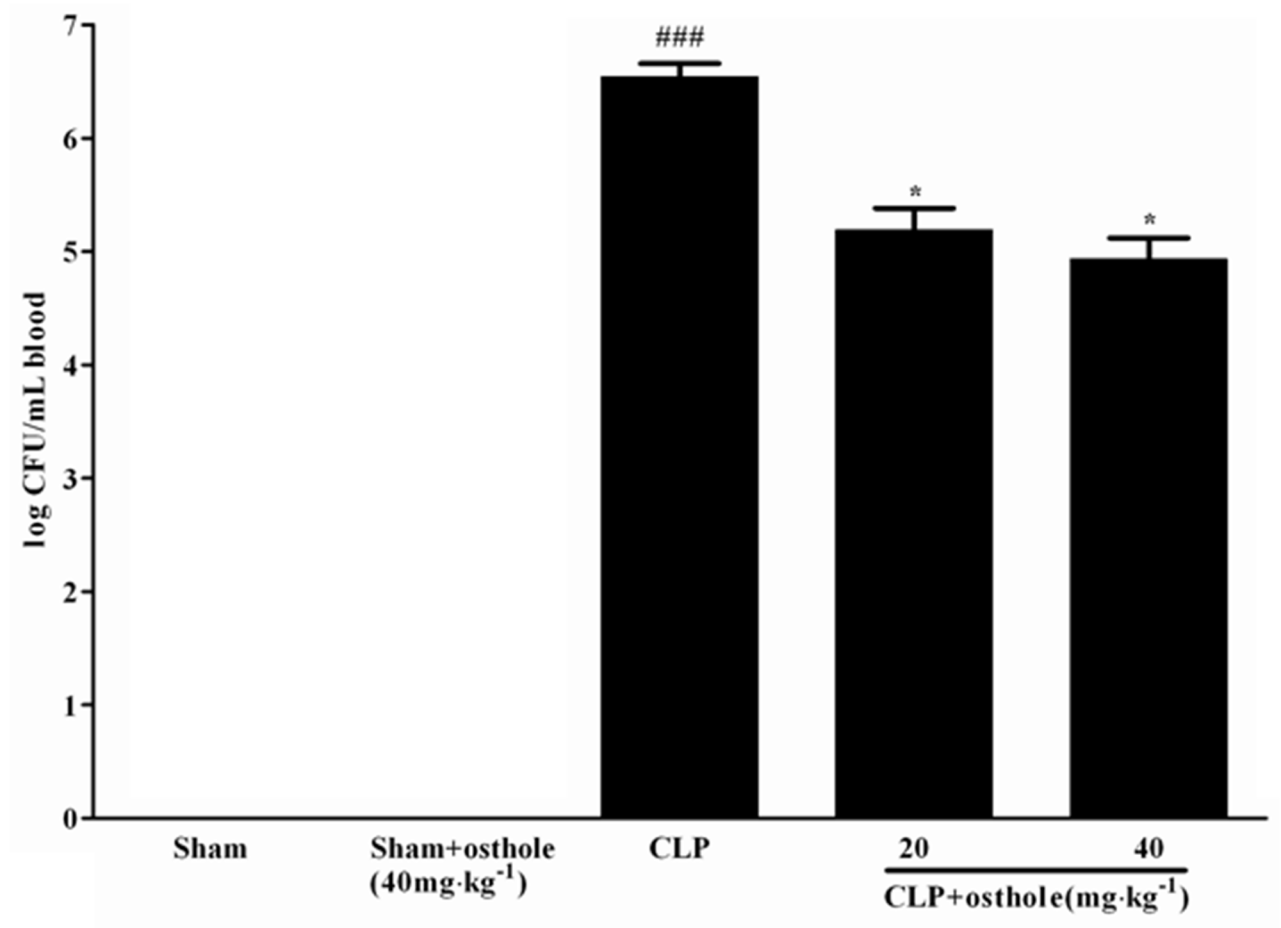
Effect of osthole on blood bacterial clearance in CLP-induced mice sepsis CLP animals were orally administrated with osthole at oral dose of 20 and 40 mg·kg^−1^. Bacteria were counted in blood 24 h after treatment. Colony-forming units (CFU) data are expressed as mean±SD. (n=10). ^###^*p<* 0.001 compared to Sham group; * *p<*0.05 compared to CLP group.

According to Figure 10, CFU was not detectable in the sham+osthole group and the sham group. The degree of CFU/mL in the CLP group was 3.50±1.08×10^6^, while CFU values in the CLP group were treated with 40mg·kg^−1^ or 20 mg·kg^−1^ of osthole and were decreased profoundly to 0.086±0.046×10^6^ and 0.16±0.088×10^6^, severally.

### Effect of osthole on killing activities of peritoneal macrophages *ex vivo*

**Figure 11:**
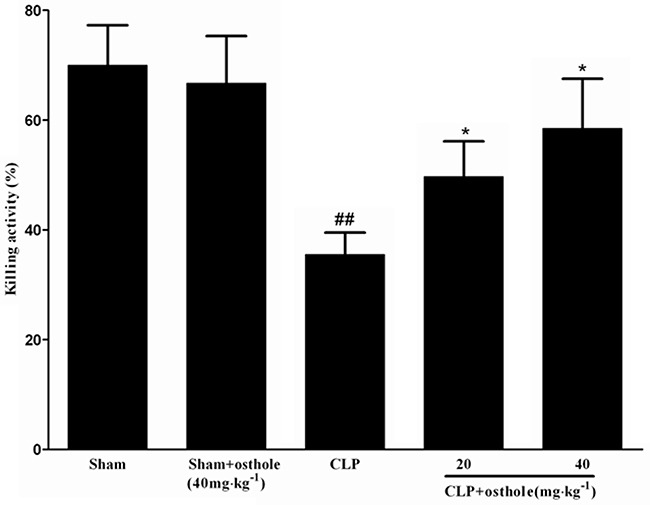
Effect of osthole on bacterial killing activity of macrophages in CLP mice CLP animals were orally administrated with osthole at oral dose of 20 and 40 mg·kg^−1^. Peritoneal macrophages were isolated and incubated with *E. coli*, followed with extensively washing and incubation in fresh medium. Then lysates were got and serially diluted to determine the CFU. Data are expressed as mean±SD. (n=10). ^##^*p<0.01* compared to Sham group; * *p<0.05 c*ompared to CLP group. CFU: Colony-forming units.

Figure 11 shows that compared with the bacterial killing activity of macrophage from sham mice, the activity from CLP mice was greatly depressed. Nonetheless, the bacterial killing activities of macrophage were increased when mice were orally administrated with osthole.

### Effect of osthole on NF-κB signal pathway

**Figure 12:**
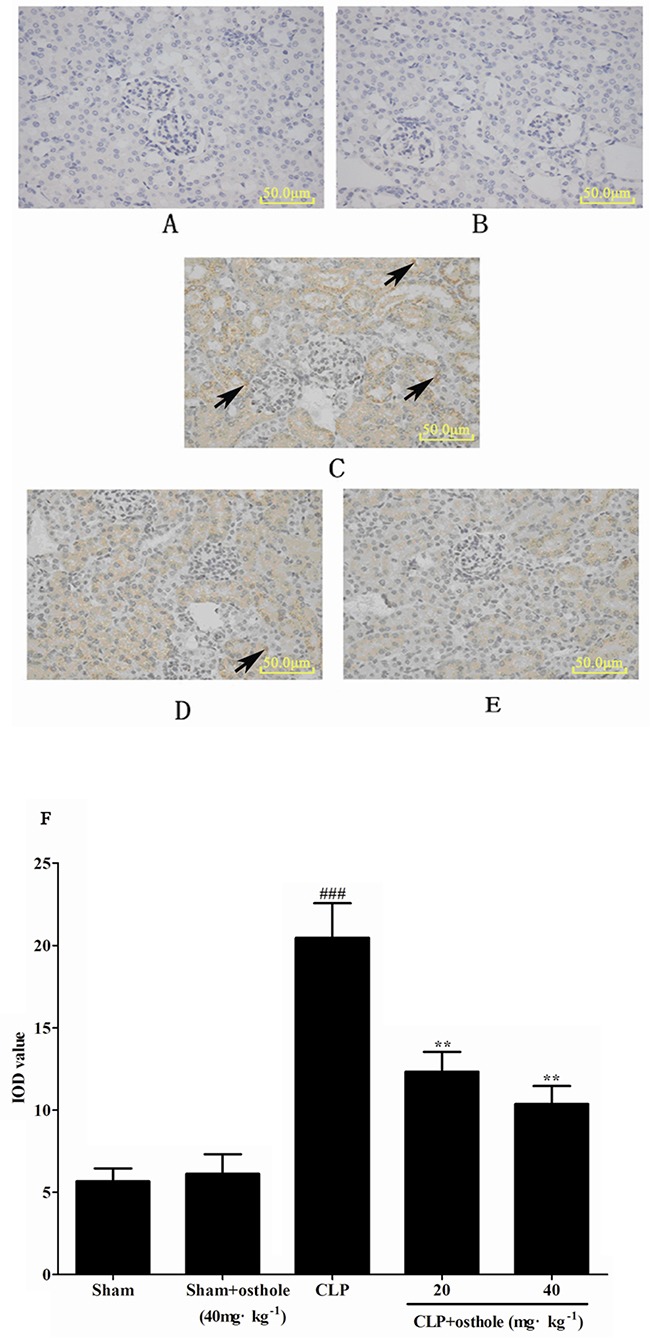
The effect of osthole on phospho-NF-κB p65 localization and expression in kidney tissue by immunohistochemistry (magnification×100) **A**. Sham group; **B**. Sham+ osthole group; **C**. CLP group; **D**. CLP + osthole (20 mg·kg^−1^) group; **E**. CLP + osthole (40 mg·kg^−1^). **F**. IOD values of phospho-NF-κB p65 staining. Data are represented as mean ± SD of 10 animals of each group, mean IOD values was measured by Image-pro plus 6.0 software. The arrowheads in the stained panels indicate positive staining. ^###^*p<* 0.001 compared to Sham group; **p< 0.01 compared to CLP group. IOD: Integral optical density.

Immunostaining for phospho-NF-κB p65 showed its localization in kidney sections. Expression of phospho-NF-κB p65 in the sham + osthole group and the sham group was barely detectable in nuclei of renal glomerulus and the proximal convoluted tubule. By contrast, increased nuclear and cytoplasmic staining for phospho-NF-κB p65 were noted in the CLP-induced group, whereas it was decreased partially by osthole treatment (Figure 12).

**Figure 13:**
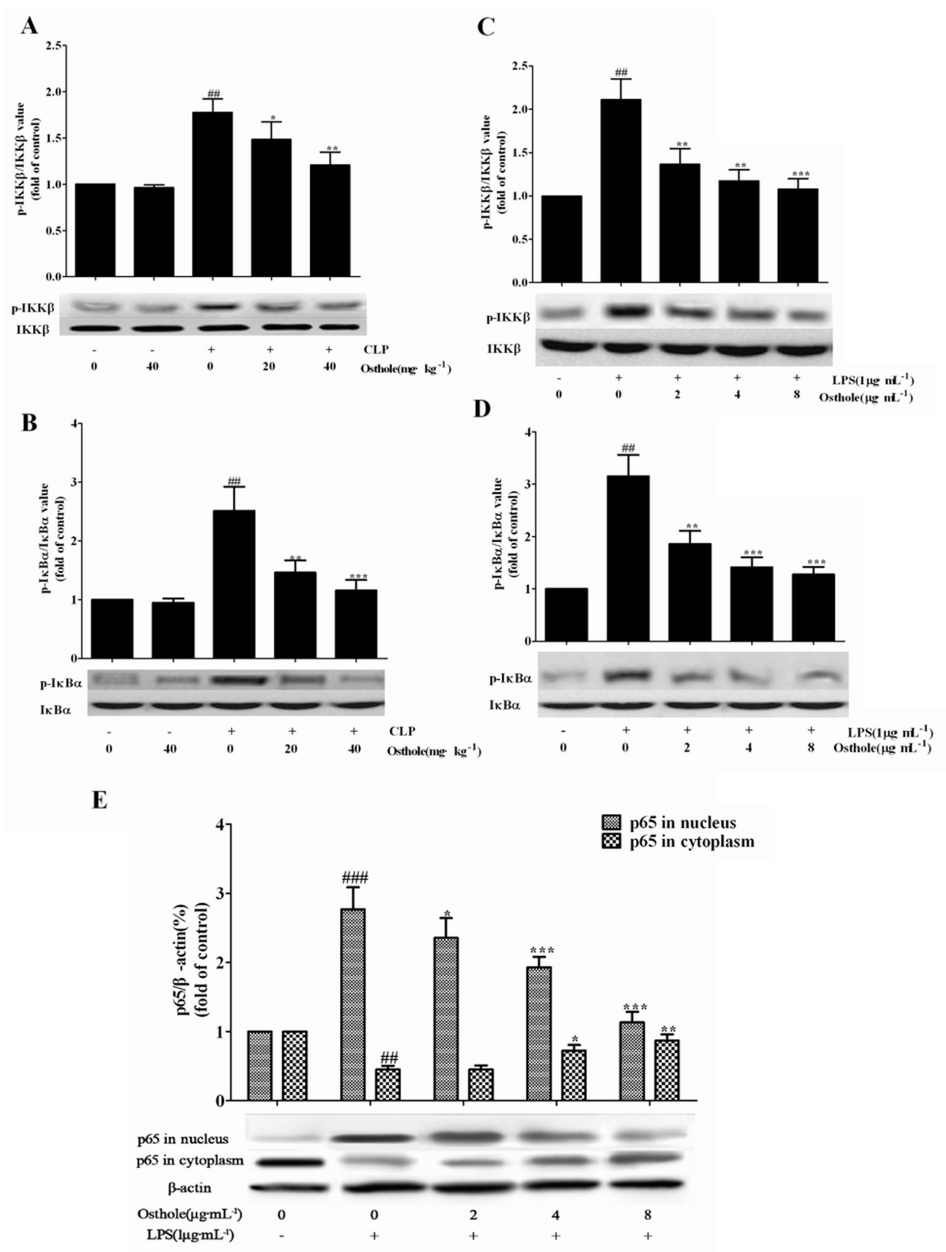
Effects of osthole on the activation of the NF-κB signalling pathway The expression of phospho-IKKβ **A**. and phospho-IκBα **B**. in kidneys from CLP-induced AKI mice (*n=*10), the expression of phospho-IKKβ **C**. and phospho-IκBα **D**. and the expression of p65 **E**. in LPS-induced RAW 264.7 cells were detected by Western blot. Data are represented as mean±SD of three independent experiments. *p< 0.05, ** *p<* 0.01 and ***p< 0.001 compared to CLP-treated mice or LPS-treated cells ; ^##^ *p<* 0.01 and ^###^*p<* 0.001 compared to sham group mice or untreated cells.

Also, the influence of osthole on the relative NF-κB signal pathway protein expression in CLP mice was investigated and RAW cells were cultured utilizing the Western blot study. Figure 13A and 13B showed that the expression of phosphorylated of IκBα and IKKβ was greatly enhanced in the CLP group in comparison to the sham group, and g the phosphorylation of IκBα and IKKβ was greatly inhibited by the osthole treatment. The phenomena could be confirmed in the culture system of *in vitro* RAW cells (Figure 13C and 13D). NF-κB p65 levels in nuclear fractions were greatly enhanced in cells induced by LPS in comparison with the control group. NF-κB p65 levels in cytoplasmic fractions were greatly decreased in cells induced by LPS in comparison with the control group. From the cytosol to the nuclear, the translocation of NF-κB p65 was greatly inhibited by incubation with various concentrations of osthole (Figure 13E).

**Figure 14:**
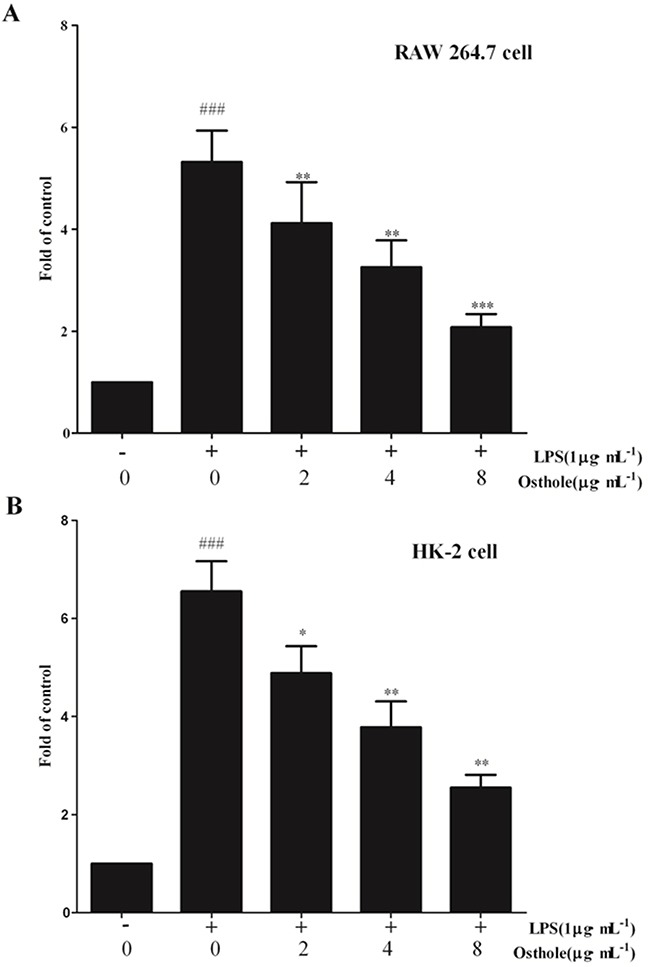
Effect of osthole on the nuclear translocation of NF-κB **A**. in RAW 264.7 cell; **B**. in HK-2 cells. The free NF-κB/p65 in nuclear extracts was assessed using the ELISA-based NF-κB kit 24 h after stimulation. Untreated group is set as 100%. Results are expressed as fold increase over untreated group. Data are represented as mean±SD of three independent experiments. ###p< 0.001 compared to untreated group, *p< 0.05, **p< 0.01 and ***p< 0.001 compared to LPS alone.

Last but not least, whether osthole could influence the nuclear translocation of NF-κB subunits by utilizing the ELISA-based NF-κB transcription factor assay kit was determined. The translocation of NF-κB p65 to nucleus (Figure 14) was strongly promoted by LPS treatment on RAW cell. However, NF-κB p65 dose induced by LPS was dependently mitigated by osthole treatment. According to our finding, activation of NF-κB signaling pathway was mitigated by osthole through inhibiting the nuclear translocation through regulation of the phosphorylation of IκBα and IKKβ.

## DISCUSSION

There is an urgent medication used to study the novel pharmacological interventions to prevent or treat AKI in consideration of the high incidence and related mortality and morbidity of sepsis-associated AKI.

The pharmacological mechanism and protective effect of osthole on acute kidney injury were examined in the current research. The cecal ligation and puncture (CLP) model used in this research was considered as superior as it shows varieties of facets of the systemic response to local infection [14]. It is similar to the mixed bacterial infection of intestinal origin in human-beings and the clinical situation of bowel perforation to study how osthole influence AKI induced by sepsis in mice [15]. The study showed that the CLP model showed a substantial kidney injury with distinct changes of serum biochemical index and histopathology of renal injury. According to our results, as an index of renal injury severity in septic AKI, osthole could attenuate the changes of histopathology, enhance the survival time greatly and decrease the rise of SCr and BUN. It shows that osthole could protect kidney injury induced by sepsis.

Excessive inflammation is of importance though the pathogenesis of AKI is not clear during septic shock [16]. Sepsis was featured by an excessive systemic pro-inflammatory response to invasive microbial pathogens. With releasing inflammatory mediators and major roles of phagocytosis, the innate immune system serves as the first line of defense against infection. Various inflammatory reactions which included migration of immune cells to the site of infection were stimulated by cytokines, which is important for infection localization. Nonetheless, during serious infection, a dysregulated cytokine release stimulated the pathophysiological abnormities of sepsis and resulted in harm to the host. Host responded to infectious challenge which led to the over expression of pro-inflammatory mediators, like IL-1β and TNF-α. The cytokines were crucial mediators in the initiating of systemic inflammatory response, which was significant in the pathogenesis of multi-system organ failure and septic shock [17]. How osthole stimulated systemic levels of pro-inflammatory cytokines was examined after CLP. The result showed that osthole greatly decreased levels of IL-6, TNF-α and IL-1β in LPS-induced RAW cells and CLP mice. Osthole can reduce the release of IL-8 and MCP-1and induced by LPS in HK-2 cells, which are significant chemoattractant for macrophages/ monocytes, T lymphocytes and neutrophils in renal inflammation. The inhibition of inflammatory mediators is likely to be regarded as a main mechanism that underlies the anti-sepsis action of osthole. In addition to the accelerating cytokine production, sepsis-induced changes in the WBC have been mentioned before and were a characteristic phenomenon during sepsis [18]. Thus, peripheral WBC counts were used to show the severity of sepsis and the migration of leucocyte. An activity of osthole was observed in attenuating the decrease of peripheral WBC. Also, colony-forming units counts in blood was correspondingly reduced, showing that neutrophil and infiltration and migration and macrophages/monocytes caused by systemic inflammatory response were ameliorated. Furthermore, at late stage of sepsis, macrophages functions were suppressed [19]. According to our research, the bacterial killing activity and phagocytic function of peritoneal macrophages was impaired partly for one day after CLP and was considerably increased after the treatment of osthole. The results as above showed that because of its immunomodulatory and anti-inflammatory features, osthole had protective effect on septic AKI.

NF-κB signal pathway is pivotal in inflammatory and immune responses. Recent researches on animal models of septic shock showed an important role of NF-κB in the pathophysiology of sepsis [20, 21]. NF-κB can be stimulated with various stimuli, such as TNF-α and LPS. Activation of NF-κB occurs through phosphorylation of the inhibitory protein IκB by translocation of NF-κB subunits to the nucleus and IκB kinases (IKKs). To induce the transcription of different target genes, it binds to DNA [22]. Activation of NF-κB signaling could be observed in cultured RAW and kidney tissue of CLP mice by detecting the phosphorylation degrees of p65 translocation and relative protein in the absence or presence of osthole treatment. The activation of NF-κB could be observed in CLP-induced septic mice and osthole considerably prevented the translocation of NF-κB p65 subunit through inhibition of the phosphorylation of p65, IκBα and IKKβ. In addition, it could be observed that *in vitro* osthole attenuated the LPS induced cell cytocotoxicity and cytokines release, like TNF--α. It could be speculated that apoptosis may be triggered by death receptor activated by cytokines release induced by LPS, whereas the process was inhibited by osthole in the dose-dependent manner.

To conclude, osthole could decrease acute kidney injury induced by sepsis. Because of immunomodulatory properties and its anti-inflammatory activity which might be associated with inhibition of the activation of nuclear translocation of NF-κB p65 and the NF-κB signal pathway through restraining the phosphorylation and expression of the relative molecule IκBα and IKKβ protein, osthole has protective effect on renal injury. According to such evidence, in acute kidney injury treatment, osthole has a potential application.

## MATERIALS AND METHODS

### Animals

BALB/c mice (weight, 18-22g) which were eight weeks old were bought from Beijing Hua Fukang Biotechnology Co. Ltd., China (certificate no. SCXK20090015). The mice were maintained with free access to tap water and standard diet and acclimated for seven days at least at humidity of 55±5% and a temperature of 24±1°C.

### Ethics statement

All the experiments of animal in our research were conducted conforming to the Guide for the Care and Use of Laboratory Animals, stipulated by the National Institutes of Health, America and approved by the local Animal Ethical Committee and Office of Experimental Animal Management Committee of Shandong Province, China on May 3^rd^, 2014.

### Reagents

Osthole (purity=98%, HPLC) was brought from Shanxi Scidoor Hi-tech Biology Co, Ltd (Batch number 20120220; Xi’An, China). NF-κB p65 ELISA kit (cat. no. NBP2-31042) was gained fromNovus Biologicals. Assay kit reagents of Serum Creatinine Determination (SCr) and Blood Urea Nitrogen (BUN) were brought from the Institute of Jiancheng Bioengineering (Nanjing, China). Sigma-Aldrich Chemical Co. (America) provided Lipopolysaccharide (LPS) from Escherichia coli 055:B5. ELISA Kits of IL-6, TNF-α, IL-1β, IL-8 and MCP-1 were brought from Yantai Science & Biotechnology (Shandong, China). Fetal bovine serum (FBS) and DMEM medium were products of Gibco Corporation (USA). Nitroblue tetrazolium (NBT), Zymosan A and the other reagents were brought from Sigma-Aldrich Chemical Co. (America). All the other reagents belonged to analytical grade were brought from Sigma-Aldrich Chemical Co. (USA).

### Cell line

Macrophage RAW 264.7 cells of mouse were brought from ScienCell Research Laboratories, America. RAW 264.7 cells were cultured in DMEM medium with 1.0% penicillin-streptomycin solution and 10% heat-inactivated FBS in a 5.0% CO_2_ humidified incubator at 37°C.

Renal proximal tubular epithelial cells (HK-2 cells) of human were brought from ScienCell Research Laboratories, America. HK-2 cells were cultured in DMEM medium with 1.0% penicillin-streptomycin solution and 10% heat-inactivated FBS in a 5.0% CO2humidified incubator at 37°C. Cells from passages 3 to 5 were utilized throughout research after recovery.

### Experimental sepsis model by CLP

Followed the original report by Baker and others [23], the CLP procedure was slightly modified to adjust to our experiment. Mice were placed in supine position and anesthetized. The abdomen was exposed to the cecum. The cecum was ligated with a 4-0 silk and sutured from the cecal tip at 5.0 mm. To spread the cecal content into the peritoneal cavity, the ligated cecal stump was punctured for two times utilizing a 22-gauge needle. 4-0 silk sutures were used to close the surgical incision. Sham-operated mice went through opening of bowel exposure and the peritoneum, yet without puncture and ligation. All the mice subcutaneously received 1 mL of normal saline for fluid resuscitation and were placed on a heating pad till recovery from anaesthesia. The mouse was returned back to a clean cage with water and food.

### Animals’ treatments

The design, similar to the previous work we did [24], was used in this study. Mice were classified into five groups randomly (n=10 mice per group): Mice in sham+osthole group went through a sham operation and were given osthole (40 mg·kg^−1^) dissolved in 0.5% CMC-Na. Mice in sham group went through a sham operation and received 0.5% sodium carboxymethycellulose (CMC-Na) as control vehicle. Mice in CLP group were exposed to CLP and received control vehicle. Mice in CLP +high dose of osthole group and CLP + low dose of osthole group were performed by CLP surgery, and administered osthole of 40 mg·kg^−1^ and 20 mg·kg^−1^severally 2 hours after operation. Blood and kidney tissue samples were gathered for quantification of biochemical analysis 24 hours after CLP.

### Survival studies

Mice exposed to CLP were assigned randomly to receive or not osthole (40 or 20 mg·kg^−1^ body weight) for evaluation of mortality rates. Sham mice went through the same process of surgery except CLP. 10 mice were contained in every group and after treatment, the mortality rates were recorded every 6 h for 3 days.

### Assessment of renal functions

At the end of the experiment, the concentrations of SCr and BUN were studied by utilizing commercial kit reagents.

### Cytokine assays

The levels of IL-8 and MCP-1 in the HK-2 supernatant and the levels of IL-6,TNF-α and IL-1β in serum from every group and in the cultural supernatants of RAW 264.7 cells could be determined by utilizing commercial ELISA kits based on the instructions of manufacturer.

### Peripheral cell counts

Blood was harvested by cardiac puncture using a heparinized syringe when the CLP experiments were completed. The white blood cell count (neutrophils, WBC; lymphocytes, monocytes) was performed utilizing an automated cell counter for murine blood cells (PE-6800VET Animal Blood Counter, San Feng Company, China).

### Determination of blood bacterial counts

Blood was aseptically collected from anesthetized BALB/c mice by cardiac puncture and diluted serially with sterile PBS. Every dilution was equally placed on plates of trypticase soy agar (TSA) and incubated overnight at 37°C. Subsequently, the amounts of aerobic bacteria colonies could be calculated.

### Phagocytosis assay of peritoneal macrophages

After CLP, Peritoneal macrophages could be harvested from peritoneal lavage fluid for a day. The cells in the peritoneal cavity of every group could be gathered. The phagocytic activity was assessed and peritoneal macrophages were isolated utilizing the assay system which has been studied before [25]. Cells were incubated with 0.5 mg·mL^−1^ of NBT and 5×10^6^ particles of opsonized zymosan. Plates were centrifuged at 4°C to pause the zymosan ingestion and supernatant was removed by flipping after 60 min of incubation. NBT reduction was colorimetrically assayed. Generally, the absorption of the formazan solution was assessed at 630 nm. The intracellular blue-black formazan deposits were solubilized with 120 μL of dimethylsulfoxide (DMSO) and 140 μL of 2M sodium hydroxide (NaOH).

### Killing activity of macrophages in vitro

Isolated peritoneal macrophages were infected with 1×10^6^ CFU of *E. coli* for 60 min. The wells were washed extensively to remove the unphagocytosed bacteria. Later, part of the wells was lysed with 0.5% Triton X-100. The cell lysates were diluted serially and put on TSA blood agar plates (Difco) and then incubated overnight at 37°C. The amounts of colonies were calculated. Another part of the wells was replaced with prewarmed fresh medium and incubated for another 12h, followed with serial dilution, culture on TSA plate and lysis for CFU count.

### Immunohistochemistry and histopathology examination

Kidney segments were embedded in paraffin sectioned (5 μm) and fixed in 10% (v/v) phosphate-buffered formalin for 24 h by utilizing a microtome 24 h after the CLP surgery. Atained with hematoxylin and eosin (H&E), it was checked by utilizing light microscopy. Five randomly selected fields for every mouse were used to estimate the level of kidney damage. Renal tubular injury was emulated by utilizing a semi quantitative score where the percentage of cortical tubules which showed epithelial necrosis was assigned a score of either 4, >75%, 3, 25-75%; 2, 10-25%; 1,<10%; or 0, none.

Kidney sections were rehydrated and dewaxed via graded ethanol solutions for immunohistochemistry and later rinsed with PBS. Endogenous peroxidase was blocked by 3% H_2_O_2_ for half an hour at 37°C in absolute methanol. The slides were incubated with primary antibody phospho-NF-κB p65 diluted to 1:200 at at 4°C for 16h in PBS, washed with PBS for three times, and then incubated for 60 min with biotin-conjugated secondary antibodies. Then the slides were washed with PBS again. Lastly, the samples used diaminobenzidine (DAB) for development. Hematoxylin was utilized as the counterstain.

### Western blotting analysis

Cells were harvested and washed with ice cold PBS for three times. Frozen kidney tissues were thawed and then homogenized mechanically to extract protein. Cytoplasmic and nuclear extracts were gained by utilizing a cytoplasmic/ nuclear isolation kit (Beyotime Institute of Biotechnology, Beijing, China). Proteins were segregated by electro-blotted and SDS-PAGE into a membrane of nitrocellulose. The blots were probed with antibodies against IκBα, IKKβ, phospho-IκBα, phospho-IKKβ and p65 (all from Cell SignalingTechnology, Beverly, MA, America) at 4°C overnight and later incubated with horseradish peroxidase-conjugated anti-IgG (Boster Biotechnology, Wuhan, Hubei province, China) as secondary antibody solution at room temperature for 60 min. The expression of every protein could be detected by the detection system of ECL (ChemiDoc™XRS, Bio-Rad). The respective bands were quantitated and images were collected utilizing Quantity One Software (Bio-Rad). The fold increase over control could be used to express the results.

### MTT assay for cell viability

The 5-diphenyl tetrazolium bromide (MTT) and 3-(4, 5-dimethyl-2-thiazolyl)-2 assay was utilized to show the cell viability. HK-2 and RAW 264.7 cells were seeded at the density of 10^4^ cells·mL^−1^in a 96-well plate. Different concentrations (2-8μg·mL^−1^) of LPS (1 μg·mL^−1^) or osthole were used to treat the cells for a day. 20 μL MTT (5 mg·mL^−1^) was added to every well and later incubated for 4 h. The formation of formazan was resolved with 150 μL per well of DMSO and the supernatant was removed. The absorbance was evaluated at 570 nm wavelength by utilizing a micro plate reader. The effect on cell proliferation was evaluated as the viability of percent cell with the control cells regarded as 100% viable.

### Measurement of nuclear translocation of NF-κB p65

HK-2 and RAW 264.7 cells were treated with different concentrations (2-8 μg·mL^−1^) of LPS (1 μg·mL^−1^) or osthole for a day. The relative rise of NF-κB p65 translocation into the nucleus was measured utilizing an ELISA kit to detect nuclear free p65 of samples after extraction of the nuclear protein utilizing the Nuclear Extract kit.

### Statistical analysis

The Kaplan–Meier method and the log rank test were used to determine the survival data. All the data were expressed as means ± SD. ANOVA could be used to determine the statistical importance of differences between groups. Differences were regarded significant at *p<* 0.05.

## Abbreviations

BUN: blood urea nitrogen
SCr: serum creatinine
TNF-α: tumor necrosis factor-α
IL-1β: interleukin-1β
IL-6: interleukin-6
IL-8: interleukin-8
AKI: Acute kidney injury
RRT: renal replacement therapies
CLP: cecal ligation and puncture
LPS: lipopolysaccharide
IKK: I kappa B kinase
ICUs: intensive care units
CFU: Colony-forming units
MCP-1: monocyte chemoattractant protein-1.
